# Immunobiotic Feed Developed with *Lactobacillus delbrueckii* subsp. *delbrueckii* TUA4408L and the Soymilk By-Product Okara Improves Health and Growth Performance in Pigs

**DOI:** 10.3390/microorganisms9050921

**Published:** 2021-04-25

**Authors:** Yoshihito Suda, Nana Sasaki, Kyoma Kagawa, Mariano Elean, Binghui Zhou, Mikado Tomokiyo, Md. Aminul Islam, Muhammad Shahid Riaz Rajoka, A. K. M. Humayun Kober, Tomoyuki Shimazu, Shintaro Egusa, Yuji Terashima, Hisashi Aso, Wakako Ikeda-Ohtsubo, Julio Villena, Haruki Kitazawa

**Affiliations:** 1 Department of Food Resource Development, School of Food Industrial Sciences, Miyagi University, Sendai 982-0215, Japan; suda@myu.ac.jp (Y.S.); f1730051@myu.ac.jp (N.S.); f1953003@myu.ac.jp (K.K.); 2 Graduate School of Food, Agricultural and Environmental Sciences, Miyagi University, Sendai 982-0215, Japan; 3 Laboratory of Immunobiotechnology, Reference Centre for Lactobacilli (CERELA-CONICET), Tucuman CP4000, Argentina; melean@cerela.org.ar; 4 Food and Feed Immunology Group, Laboratory of Animal Food Function, Graduate School of Agricultural Science, Tohoku University, Sendai 980-8572, Japan; zhou.binghui.s5@dc.tohoku.ac.jp (B.Z.); mikado.tomokiyo.t4@dc.tohoku.ac.jp (M.T.); aminul.vmed@bau.edu.bd (M.A.I.); shahidrajoka@yahoo.com (M.S.R.R.); humayuna2002@yahoo.com (A.K.M.H.K.); wakako.ohtsubo.a7@tohoku.ac.jp (W.I.-O.); 5 Livestock Immunology Unit, International Education and Research Center for Food and Agricultural Immunology (CFAI), Graduate School of Agricultural Science, Tohoku University, Sendai 980-8572, Japan; hisashi.aso.a6@tohoku.ac.jp; 6 Department of Medicine, Faculty of Veterinary Science, Bangladesh Agricultural University, Mymensingh-2202, Bangladesh; 7 Department of Dairy and Poultry Science, Faculty of Veterinary Medicine, Chittagong Veterinary and Animal Sciences University, Khulshi, Chittagong 4225, Bangladesh; 8 Department of Food Science and Business, School of Food Industrial Sciences, Miyagi University, Sendai 982-0215, Japan; shimadut@myu.ac.jp; 9 Research and Development Division, Marusan-Ai Co., Ltd., Okazaki 444-2193, Japan; shitaro.egusa@marusanai.co.jp (S.E.); yuji.terashima@marusanai.co.jp (Y.T.); 10 Laboratory of Animal Health Science, Graduate School of Agricultural Science, Tohoku University, Sendai 980-8572, Japan

**Keywords:** okara, soymilk by-product, *Lactobacillus delbrueckii* subsp. *delbrueckii* TUA4408L, probiotics for pigs, immunobiotics, pig performance, pig immune health

## Abstract

*Lactobacillus delbrueckii* subsp. *delbrueckii* TUA4408L is able to differentially modulate the innate immune response of porcine intestinal epithelial cells triggered by TLR4 activation. This strain also has a remarkable ability to grow on plant substrates. These two immunological and biotechnological characteristics prompted us to evaluate whether the soymilk by-product okara fermented with the TUA4408L strain can serve as an immunobiotic feed with the ability to beneficially modulate the intestinal immunity of piglets after weaning to improve their productivity. Our in vivo studies demonstrated that the administration of immunobiotic TUA4408L-fermented okara feed significantly increased piglet growth performance and meat quality. These positive effects were associated with the ability of the TUA4408L-fermented okara feed to beneficially modulate both intestinal microbiota and immunity in pigs. The immunobiotic feed improved the abundance of the beneficial bacteria *Lactobacillus* and *Lactococcus* in the gut of pigs, reduced blood markers of inflammation, and differentially regulated the expression of inflammatory and regulatory cytokines in the intestinal mucosa. These findings indicate that the immunobiotic TUA4408L-fermented okara feed could be an economical and environmentally friendly option to improve the growth performance and immune health of pigs.

## 1. Introduction

The early events in a pig’s life are of extreme importance for productivity. Alterations in this stage of life that result in growth lag can increase barn occupancy and diminish market weights, imposing a significant cost to the productive system [1]. It has been established that the growth performance of piglets depends on several factors that include age at weaning, management procedures, and pathogenic load in the pig environment. Thus, strategies able to increase the welfare and growth performance of pigs at weaning have been extensively researched in the last decades [1]. The management strategies included dietary interventions aimed at modulating pigs’ oxidative and inflammation status. In this regard, probiotic microorganisms have been proposed as an alternative to avoid non-protective inflammation and to improve resistance against intestinal infections in piglets. These immunomodulatory probiotics or immunobiotics have been shown to positively influence the maturation of the immune system, the defenses against pathogens, and the control of detrimental inflammation [2,3]. Hence, the inclusion of immunobiotics in feed supplementation holds promise as a way to exert positive effects on weanling pigs [4].

The white-yellowish puree “okara” is a by-product of soymilk production that is obtained after the filtration of pulverized soybeans [5]. Okara is obtained in large quantities during the production of soymilk. It is estimated that 1.1 kg of okara is obtained after the processing of 1 kg of soybean [6]. Classically, okara was considered an agro-waste product and therefore, it was dumped as landfill or burned as waste, which entailed a significant disposal problem [7]. However, in recent years this soymilk by-product has come to be considered as an interesting substrate for other biotechnological applications. Due to its abundance of dietary fiber, unsaturated lipids, high-quality proteins, and minerals [8,9], okara has been proposed for use as animal feed. On the other hand, okara has also been suggested as a cost-effective culture medium for the production of large quantities of starters or probiotics at an industrial level [10,11,12]. These characteristics of okara make it an interesting alternative for the development of a probiotic or immunobiotic feed with the ability to improve the health and productivity of pigs.

The capacity of probiotic or immunobiotic microorganisms to interact with and modulate the cellular responses of intestinal epithelial cells (IECs) has been proposed as one of the most important mechanisms involved in the protection against pathogens. The probiotic-IEC interactions have been associated with the strengthening of the epithelial barrier and the beneficial modulation of the immune system of the intestinal mucosa [2,13,14]. Moreover, the ability of immunobiotic bacteria to modulate the signaling pathways triggered by the activation of pattern recognition receptors (PRRs) such as toll-like receptors (TLRs) in IECs has been shown to be important to improved resistance against infections [2]. In this regard, we have used a porcine intestinal epitheliocyte (PIE) cell line to screen and select potent immunomodulatory lactobacilli to combat infections and to study the molecular mechanisms involved in their beneficial effects [2,13]. Among the immunobiotic lactobacilli strains evaluated by our group, the plant derived *Lactobacillus delbrueckii* subsp. *delbrueckii* TUA4408L was able to differentially modulate the innate immune response of PIE cells to the challenge with enterotoxigenic *Escherichia coli* (ETEC) [15]. In our hands, the TUA4408L strain was capable of reducing expression of the pro-inflammatory cytokines IL-6, IL-8, and MCP-1 in ETEC-challenged PIE cells. The immunoregulatory ability of *L. delbrueckii* subsp. *delbrueckii* TUA4408L was associated with a reduction in the activation of the NF-κB, ERK-MAPK, and p38-MAPK pathways. Our studies in PIE cells also revealed that TLR2 and the negative regulators of the TLR signaling pathway play key roles in the immunomodulatory activity of the TUA4408L strain. This immunobiotic bacterium significantly increased the expression of SIGIRR and Tollip in PIE cells, and the knockdown of TLR2 in PIE cells eliminated the ability of *L. delbrueckii* subsp. *delbrueckii* TUA4408L to up-regulate the TLR negative regulators and differentially modulate the inflammatory response [15].

Our previous studies of PIE cells clearly demonstrated the immunoregulatory potential of *L. delbrueckii* subsp. *delbrueckii* TUA4408L. However, these in vitro models are simplified and may neglect the effect of cell-cell interactions in the complex intestinal microenvironment, which could change the resulting response. Hence, in order to accurately demonstrate the immunomodulatory capacity of the TUA4408L strain, in vivo studies were mandatory. Thus, in this work we aimed to investigate whether the in vitro immunomodulatory effects of *L. delbrueckii* subsp. *delbrueckii* TUA4408L reported in our previously published works could also be found in vivo. Moreover, taking into consideration the remarkable ability of the TUA4408L strain to grow on plant substrates [16], we evaluated here whether the *L. delbrueckii* subsp. *delbrueckii* TUA4408L-fermented okara can serve as an immunobiotic feed with the ability to beneficially modulate the intestinal immunity of piglets after weaning to improve their immune health status and productivity.

## 2. Materials and Methods

### 2.1. L. delbrueckii subsp. delbrueckii TUA4408L and Fermentation of Soymilk By-Product

*L. delbrueckii* subsp. *delbrueckii* TUA4408L was isolated from sunki, a Japanese traditional pickle fermented in salt-free condition, as described previously [15,16]. The TUA4408L strain was maintained in deMan-Rogosa-Sharp (MRS) medium at 4 °C. *L. delbrueckii* subsp. *delbrueckii* TUA4408L was propagated in soymilk at 43 °C for 16 h.

### 2.2. Animals and Treatments

This study was carried out in strict accordance with the recommendations in the Guide for the Care and Use of Laboratory Animals of the Guidelines for Animal Experimentation of Miyagi University, Sendai, Japan. The Laboratory Health and Safety Committee of Miyagi University approved the present study, with a permitted protocol No. 2016-23. All efforts were made to minimize suffering.

Pig were produced by crossbreeding (LWD) with Landrace (L), Large Yorkshire (W), and Duroc (D). Animals were allocated to 3 groups of 5 heads each: untreated pigs (control group), pig fed with soymilk (SM control group), and pigs fed with the *L. delbrueckii* subsp. *delbrueckii* TUA4408L-fermented okara (TUA4408L group). Okara contains several anti-nutritional factors and toxic compounds such as antitrypsin, saponin, hemagglutinin, and trypsin inhibitor; thus, it can affect the digestion and absorption of nutrients in pigs [17]. In addition, it was demonstrated that dry okara significantly reduced meat quality, especially the juiciness and the color of pork [18]. Therefore, soymilk, not unfermented okara or dried okara, was chosen as control.

Piglets were taken from the same or closely related litters to exclude a family effect. After weaning, all pigs were raised and fattened with the administration of 3 kinds of commercial diets. Each diet was administered to the 3 groups of pigs after the animals reached a preset weight. Nutrient compositions of the diets are shown in Table S1. The nutrient components were arranged by mixing corn, wheat, milo, soybean meal, rapeseed, rice bran, animal fats and oils, calcium carbonate, calcium phosphate, and salt. In the case of supplementation with soymilk or the fermented *L. delbrueckii* subsp. *delbrueckii* TUA4408L-fermented okara, the other components of the diets were adjusted in order to have the same percentages of proteins, fats, and calories.

Body weight measurement was carried out every week. Stool and blood samples were taken at the indicated time points. Plasma separated from blood and fresh stool samples were stored at −20 °C until analysis. Carcasses were also evaluated after the sacrifice of the animals.

### 2.3. Evaluation of Meat Quality

The evaluation of carcass characteristics and meat quality was performed as described before [19]. The standards of the Japanese Meat Grading Association were used for carcass grading evaluation. Carcass meats were judged as high, middle, or low grades.

### 2.4. Evaluation of Meat Lipids

The fatty acid composition of subcutaneous fat was evaluated in cross-sections of longissimus muscle. Loin core was analyzed by a gas chromatograph (G-6000 type, Hitachi High-Tech Global, Tokyo, Japan). The extraction of total lipids from tissues and the determination of their concentrations were carried out as described previously by our group [20].

### 2.5. Plasma Determinations

Plasma concentrations of triglycerides, cholesterol, and HDL were assessed with the Fujifilm clinical chemical analyzer (Fujifilm Dri-Chem 3500i, the Fujifilm Dri-Chem Slides, Tokyo, Japan), following the standard protocol as described previously [19]. Plasma C reactive protein (CRP) concentration was assessed with the porcine C Reactive Protein ELISA kit (Croud-Clone Corp., Wuhan, China), following the instructions of the supplier.

### 2.6. Blood Leukocytes

Blood leukocyte counts were assessed with a Celltac MEK-4100 (Nihonkohden Co.ltd., Tokyo, Japan) and specific buffers, as described previously [19]. The granulocyte/lymphocyte ratio in peripheral blood was evaluated by determining the percentage of each leucocyte population in smear preparations of peripheral blood samples. Smear preparations were made using Diff-Qick stain solution. Repeated counts of 3 times per 1 smear preparation were performed using light microscope.

### 2.7. Detection of Pathogenic Escherichia coli in Feces

In order to detect pathogenic *Escherichia coli* in stools, Western blotting was performed using anti-ETEC K88 and anti-ETEC K99 fimbrial antisera (#SSI51172, SSI51173, Veritas Co., Tokyo, Japan), and anti-ETEC 987P fimbrial antisera (originally generated in rabbit immunized with purified pili of ETEC987P) for determination of specific pili. Horseradish peroxidase conjugated anti-rabbit IgG was used as secondary antibody (#7074, Cell Signaling Technology Japan, K.K., Tokyo, Japan). All procedures were followed according to the instructions of the commercial kit, ECL Western Blotting Detection System (GE Healthcare). Feces samples were agitated by sonication and separated by centrifugation for 5 min at 20 °C. The precipitate was dissolved by using Thermo Scientific Tissue Protein Extraction (T-PER) Reagent (Tokyo), and purified by centrifugation. The supernatant was used for the detection of pathogenic *E. coli* in stools.

### 2.8. Intestinal Microbiota

The intestinal microbiota were analyzed by the T-RFLP method. In this method, the 16s ribosomal RNA is PCR-amplified with a terminal fluorescently labeled primer, cleaved with restriction enzymes *Msp*I and *Alu*I, and subjected to capillary electrophoresis for fragment analysis. The PCR method with Takara Ex Taq (Takara Bio, Tokyo, Japan) was carried out by supplying DNA from fecal samples of pigs to amplify a part of the 16S rRNA gene fragment. The universal primers 27F (5′-AGAGTTTGATCCTGGCTCAG-3′) and 1492R (5′-GGTTACCTTGTTACGACTT-3′) were used for the PCR. The 27F primer was modified by 6-carboxyfluorescein at the 5′ end. After purification, the PCR products were digested with *Msp*I and *Alu*I enzymes. Lengths of terminal restriction fragments included in the digested PCR products were analyzed by the ABI PRISM 310 genetic analyzer (Applied Biosystems, Foster City, CA, USA) according to [21]. Then, OUTs were detected on the basis of the lengths of the terminal restriction fragments’ profiles [22].

### 2.9. Immune Factor Expression in Intestinal Tissue

The expression of IFN-γ, MCP-1, IL-6, IL-8, IL-10, and TGF-β in intestinal tissues was determined by RT-qPCR. The TB Green Premix Ex Taq II of TAKARA Bio and Thermal Cycler Dice Real Time PCR system II was used as described previously [19]. The GAPDH gene was used as a reference gene. The expression of GAPDH was used to normalize cDNA levels for differences in total cDNA levels in the samples. Primers were as described previously [15,23,24].

### 2.10. Statistical Analysis

Statistical analysis was performed using the SAS program (v.9.1). Relative indices were calculated respectively as the ratio of cytokine mRNA expression to GADPH. Relative indices were respectively normalized by common logarithmic transformation and confirmed as approximate value included significantly into normal distribution. To examine the significance for a fixed effect among experimental conditions, one-way ANOVA was carried out. Duncan’s method for multi-comparison was then performed to compare among means of every category at 5% or 1% significance level.

## 3. Results

### 3.1. Immunobiotic TUA4408L-Fermented Okara Feed Improves Growth Performance and Meat Quality of Pigs

**Figure 1:**
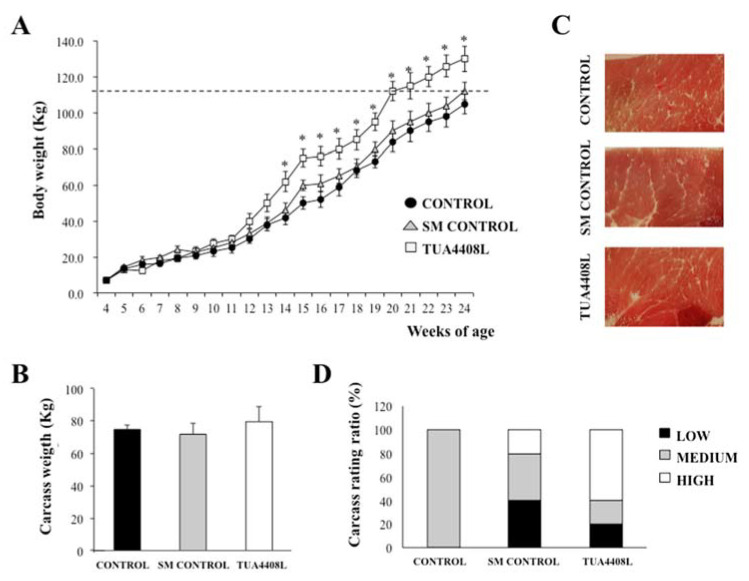
Effect of the immunobiotic TUA4408L-fermented okara feed on piglet growth and carcass quality. Pigs were grown from 3 weeks of age until week 24. Five pigs were used for each experimental group. The control group was fed only the different balanced conventional diets without antimicrobials while the SM control group and the TUA4408L group were fed balanced conventional diets with supplemental soymilk or the immunobiotic TUA4408L-fermented okara, respectively. (**A**) Body weight changes were recorded weekly. The line in body weight indicates the suitable body weight for shipping in Japan. (**B**) Carcass weight, (**C**) macroscopic analysis of carcass, and (**D**) carcass rating ratio were evaluated after sacrifice. Carcass meats were judged as high, middle or low grades. Asterisks (*) indicate statistical differences between the treated and control groups (*p* < 0.05).

We first evaluated the effect of the immunobiotic TUA4408L-fermented okara feed on the growth performance of pigs. For this purpose, we followed the body weight changes from week 4 until animals reached the weight of 115 kg (Figure 1A). Control pigs reached the weight of 115 kg in week 24, which was expected according to our previous studies [19]. Similarly, pigs in the SM control group needed the same period of time to reach the weight used as the end point. In contrast, pigs treated with the immunobiotic TUA4408L-fermented okara feed reached the weight of 115 kg in week 20, 4 weeks earlier than both control groups.

In addition, we evaluated carcass weight in the three groups of pigs as shown in Figure 1B. No significant differences were observed in carcass weight when controls were compared to pigs under treatment with SM or the immunobiotic TUA4408L-fermented okara feed. However, macroscopic analysis of the meat, particularly the loin, indicated that the immunobiotic TUA4408L-fermented okara feed increased the intramuscular fat content (Figure 1C), which could produce a meat with more softness. Then, we performed a carcass grading evaluation according to the standards of the Japanese Meat Grading Association as described previously [19]. In the control group, 100% (5 of 5 animals) of the carcasses corresponded to medium-grade quality, while in the group treated with SM, we found 20% (1 of 5 animals) and 40% (2 of 5 animals) of the carcasses to be of high- and medium-grade quality, respectively (Figure 1D). Of note, in the immunobiotic TUA4408L-fermented okara feed group, the medium-grade carcass quality represented 20% (1 of 5 animals), while the high-grade quality reached a value of 60% (3 of 5 animals). Surprisingly, 40% (2 of 5 animals) and 20% (1 of 5 animals) in the SM and the immunobiotic TUA4408L-fermented okara feed groups, respectively, had low-grade carcass quality (Figure 1D). Next, in order to investigate the composition of subcutaneous fat of the loin core, meat samples were methyl esterified and gas chromatography was used to evaluate several fatty acids (Table 1). A significant reduction in myristic acid was observed in both the SM and immunobiotic TUA4408L-fermented okara feed groups when compared to the controls. In addition, the immunobiotic TUA4408L-fermented okara feed treatment was capable of decreasing the levels of stearic acid and other saturated fatty acids and increasing the levels of linoleic acid and other unsaturated fatty acids.

**Figure 2:**
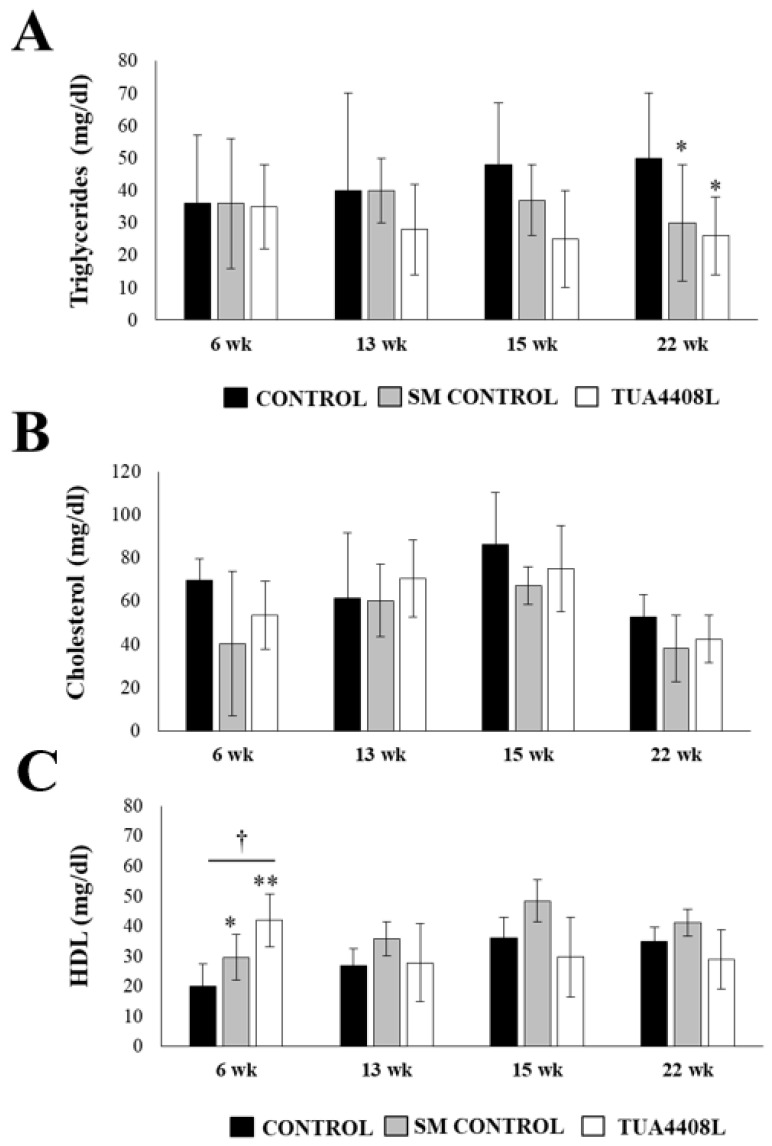
Effect of the immunobiotic TUA4408L-fermented okara feed on piglets’ plasma lipids. Pigs were grown from 3 weeks of age until week 24. Five pigs were used for each experimental group. The control group was fed only the different balanced conventional diets without antimicrobials, whereas the SM control group and the TUA4408L group were fed balanced conventional diets with supplemental soymilk or the immunobiotic TUA4408L-fermented okara feed, respectively. (**A**) Plasma triglycerides, (**B**) plasma cholesterol and, (**C**) plasma HDL were determined on weeks 6, 13, 15 and 22. Asterisks indicate statistical differences between treated and control groups (* *p* < 0.05), (** *p* < 0.01). Symbol (†) indicates statistical differences between the SM control and TUA4408L groups (*p* < 0.05).

Taking into consideration that the immunobiotic TUA4408L-fermented okara feed treatment was capable of changing the composition of the meat fat content, we aimed to evaluate whether this treatment influenced the blood lipids in pigs. Therefore, we studied the levels of plasma triglycerides, cholesterol, and HDL in pigs in weeks 6, 13, 15, and 22 (Figure 2). High variability between individuals of the same group was found during study of the three parameters. No significant differences between control pigs and animals treated with SM or the immunobiotic TUA4408L-fermented okara feed were found by the analyses of blood triglycerides at weeks 6, 13, or 15 (Figure 2A). However, for both treatment groups, this parameter was reduced at week 22 when compared to the untreated controls. Blood cholesterol studies detected no differences between the groups at any time points (Figure 2B). In addition, no differences in blood HDL were found between the groups with the exception of week 6, in which both the SM and the immunobiotic TUA4408L-fermented okara feed treatment groups had higher levels of this parameter (Figure 2C). Of note, the blood HDL levels in the immunobiotic TUA4408L-fermented okara feed group were significantly higher than observed in the SM group.

### 3.2. Immunobiotic TUA4408L-Fermented Okara Feed Improves the Health of Pigs

The improved growth performance and meat quality in pig production has been associated with good health status, particularly enhanced resistance to infections [2,3,19]. Hence, we studied the presence of symptoms associated with infectious diseases in the three experimental groups (Table 2). In control pigs, we detected the presence of both intestinal and respiratory infections during the studied period. Moreover, cases of skin, kidney, and liver diseases were also found in this group of animals. In the pigs of the SM control group, we also detected cases of intestinal and respiratory infections. Of note, no signs of infectious or other disease were found in the group of pigs treated with the immunobiotic TUA4408L-fermented okara feed, with the exception of one case of mild diarrhea (Table 2).

**Figure 3:**
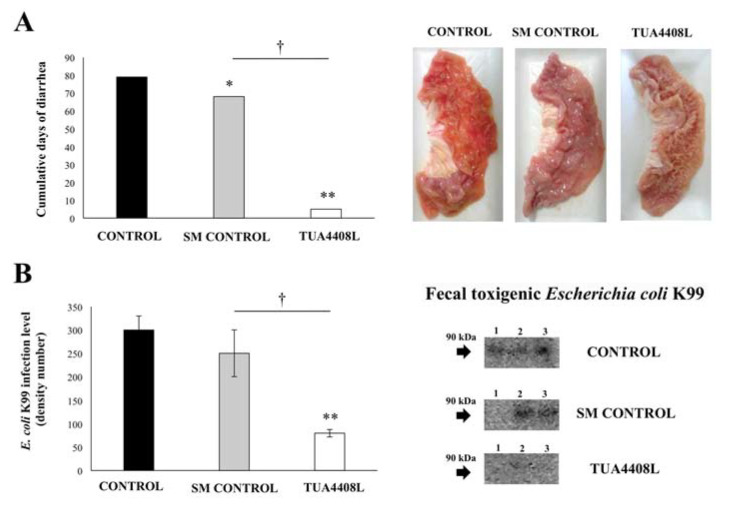
Effect of the immunobiotic TUA4408L-fermented okara feed on piglets’ health. Pigs were grown from 3 weeks of age until week 24. Five pigs were used for each experimental group. The control group was fed only the different balanced conventional diets without antimicrobials, while the SM control group and the TUA4408L group were fed balanced conventional diets with supplemental soymilk or the immunobiotic TUA4408L-fermented okara feed, respectively. (**A**) Cumulative days of diarrhea and intestinal macroscopic analysis, and (**B**) detection of enterotoxigenic *Escherichia coli* in feces were determined when there were signs of intestinal infection. Detection of pathogenic *E. coli* was performed by Western blotting method using anti-ETEC K99 fimbrial antisera in fecal samples. Asterisks indicate statistical differences between treated and control groups (* *p* < 0.05), (** *p* < 0.01). Symbol (†) indicates statistical differences between the SM control and TUA4408L groups (*p* < 0.05).

Because we were particularly interested in the incidence and severity of intestinal infections, we recorded the days of diarrhea in each group of pigs. As shown in Figure 3A, the cumulative days of diarrhea in the control pigs reached a value of 79 whereas this parameter reached 68 in the pigs treated with SM, with a statistically significant difference between both groups. The immunobiotic TUA4408L-fermented okara feed group showed a value of 5 when the cumulative days of diarrhea were evaluated, which represents a marked difference with the control groups.

In line with these findings, the macroscopic analysis of the intestinal mucosa showed significantly higher damage in the control pigs than in the immunobiotic TUA4408L-fermented okara feed group (Figure 3A). Furthermore, the analysis of the presence of ETEC K99 in pigs’ feces at week 8 using the Western blotting method and anti-fimbrial antisera demonstrated a significant reduction in the presence of this pathogenic *E. coli* in the immunobiotic TUA4408L-fermented okara feed group when compared to the controls and SM-treated pigs (Figure 3B). No ETEC K88 or ETEC 987P were detected in any of the experimental groups during the studied period (data not shown).

### 3.3. Immunobiotic TUA4408L-Fermented Okara Feed Modulates the Composition of the Intestinal Microbiota of Pigs

**Figure 4:**
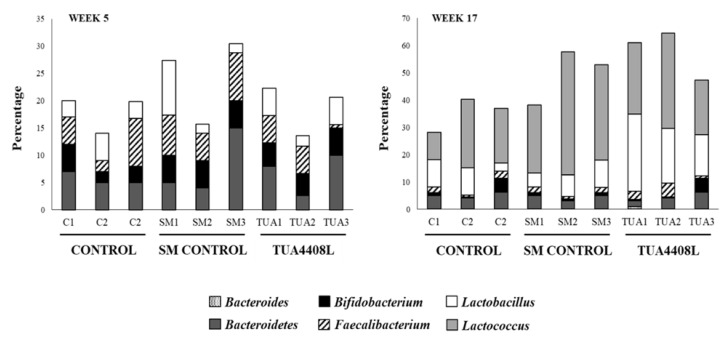
Effect of the immunobiotic TUA4408L-fermented okara feed on piglets’ intestinal microbiota. Pigs were grown from 3 weeks of age until week 24. Five pigs were used for each experimental group. The control group was fed only the different balanced conventional diets without antimicrobials, while the SM control group and the TUA4408L group were fed balanced conventional diets with supplemental soymilk or the immunobiotic TUA4408L-fermented okara feed, respectively. The composition of the intestinal microbiota was analyzed in fecal samples in weeks 5 and 17 by the T-RFLP method.

We next evaluated the effect of the feed treatments on the composition of the intestinal microbiota of pigs. For this purpose, we used the T-RFLP method to study porcine intestinal microbiota at weeks 5 and 17 (Figure 4). In young (5-week-old) pigs, the most abundant microorganisms detected in the intestine belonged to the groups of *Bacteroidetes* and *Faecalibacterium* with lower proportions of *Bifidobacterium* and *Lactobacillus*. Although a trend was observed in the increase of *Bifidobacterium* and *Lactobacillus* for both SM and the immunobiotic TUA4408L-fermented okara feed groups, the differences were not statistically significant. When 5-week-old and 17-week-old pigs were compared, a clear age-dependent increase in the *Lactococcus* group was observed in all experimental groups (Figure 4). No significant differences were found in the intestinal microbiota composition when control and SM groups were compared, with the exception of the higher percentage of lactocci in the last group. In addition, the 17-week-old pigs in the immunobiotic TUA4408L-fermented okara feed group showed significantly higher percentages of both *Lactococcus* and *Lactobacillus* when compared to the controls (Figure 4).

### 3.4. Immunobiotic TUA4408L-Fermented Okara Feed Modulates the Immunity of Pigs

**Figure 5:**
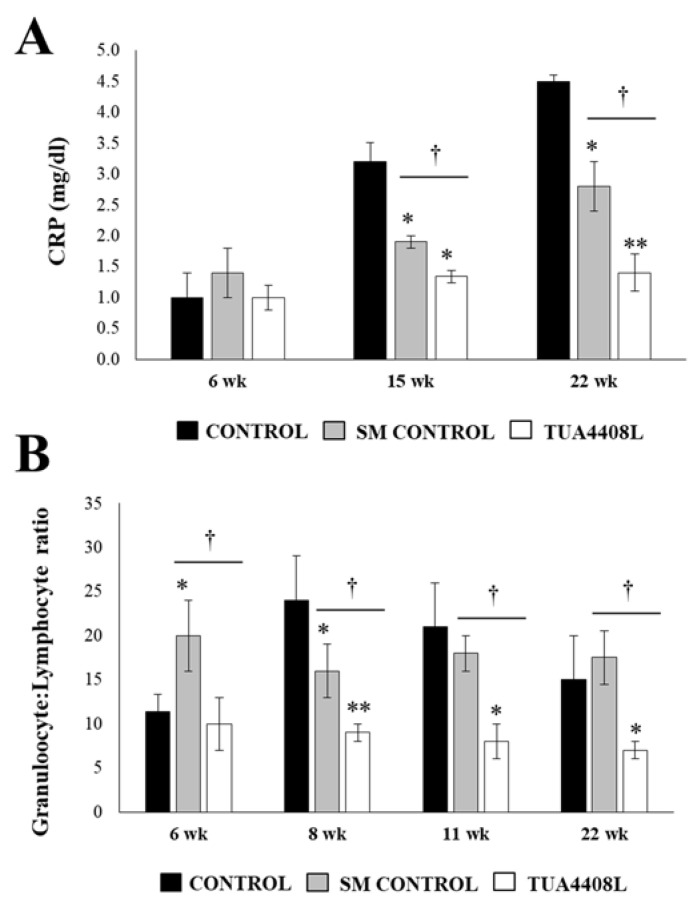
Effect of the immunobiotic TUA4408L-fermented okara feed on piglets’ immune health. Pigs were grown from 3 weeks of age until week 24. Five pigs were used for each experimental group. The control group was fed only the different balanced conventional diets without antimicrobials, while the SM control group and the TUA4408L group were fed balanced conventional diets with supplemental soymilk or the immunobiotic TUA4408L-fermented okara feed, respectively. (**A**) C reactive protein (CRP) and (**B**) the granulocyte/lymphocyte ratio were determined in weeks 6, 8, 11, and 22. Asterisks indicate statistical differences between treated and control groups (* *p* < 0.05), (** *p* < 0.01). Symbol (†) indicates statistical differences between the SM control and TUA4408L groups (*p* < 0.05).

Our previous reports evaluating the immunomodulatory activities of *L. delbrueckii* subsp. *delbrueckii* TUA4408L demonstrated its high potential to modulate the porcine immune system [15,16]. Therefore, we next aimed to determine whether the beneficial effects observed in growth and meat quality induced by the TUA4408L strain found in this study were related to changes in the immune system of pigs. The C reactive protein (CRP) levels were determined in plasma at weeks 6, 15, and 22 (Figure 5A). An age-dependent increase of CRP was observed in all the experimental groups. No differences in levels of plasma CRP were detected between the groups at week 6. However, both the SM and the immunobiotic TUA4408L-fermented okara feed groups had significantly lower levels of CRP at weeks 15 and 22.

Of note, the immunobiotic TUA4408L-fermented okara feed was more effective than the SM treatment in reducing the plasma values of CRP (Figure 5A). In addition, we evaluated the blood granulocyte/lymphocyte ratio (Figure 5B). The treatment of pigs with SM induced a significantly higher and lower granulocyte/lymphocyte ratio at weeks 6 and 8, respectively, when compared to control pigs. At weeks 11 and 22, these two groups did not show differences in this parameter. Although no differences were detected between the control and immunobiotic TUA4408L-fermented okara feed groups when the granulocyte/lymphocyte ratio was studied at week 6, the pigs treated with the TUA4408L strain had significantly lower levels of this parameter in weeks 8, 11, and 22 (Figure 5B).

**Figure 6:**
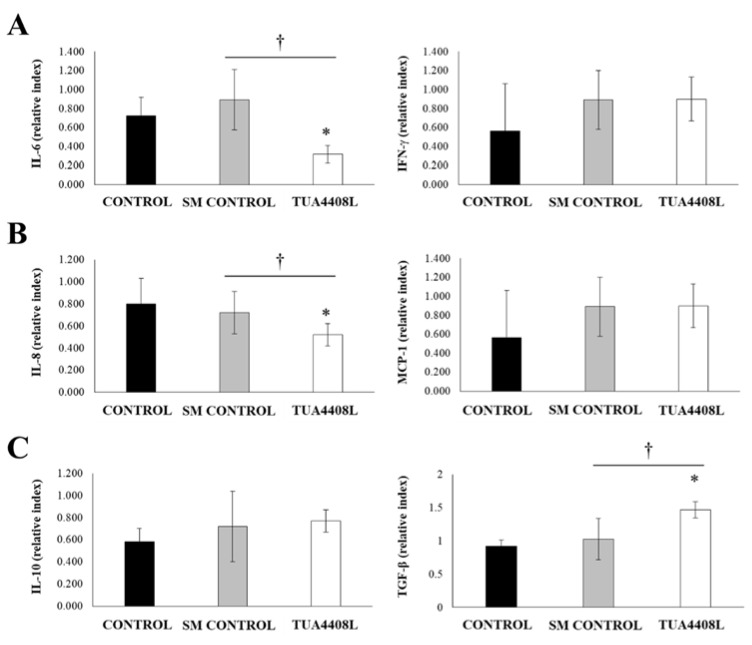
Effect of the immunobiotic TUA4408L-fermented okara feed on piglets’ immune health. Pigs were grown from 3 weeks of age until week 24. Five pigs were used for each experimental group. The control group was fed only the different balanced conventional diets without antimicrobials, while the SM control group and the TUA4408L group were fed balanced conventional diets with supplemental soymilk or the immunobiotic TUA4408L-fermented okara feed, respectively. The expression of (**A**) inflammatory cytokines (IFN-γ and IL-6), (**B**) chemokines (MCP-1 and IL-8), and (**C**) regulatory cytokines (IL-10 and TGF-β) were determined in intestinal tissues after sacrifice. Asterisks (*) indicate statistical differences between treated and control groups (*p* < 0.05). Symbol (†) indicates statistical differences between the SM control and TUA4408L groups (*p* < 0.05).

Finally, we evaluated the expression of inflammatory cytokines (Figure 6A), chemokines (Figure 6B), and regulatory cytokines (Figure 6C) in the intestine of pigs. No differences between the groups were observed when the intestinal expression of IFN-γ (Figure 6A) or MCP-1 (Figure 6B) were evaluated. In addition, no differences were detected in the expression of IL-6 or IL-8 between the control and SM groups. However, the pigs in the immunobiotic TUA4408L-fermented okara feed group had significantly lower expression of IL-6 and IL-8 than the control groups (Figure 6A,B). The expression levels of intestinal IL-10 were similar in all the experimental groups. In addition, intestinal TGF-β expression was not different when the control and SM groups were compared. In contrast, the pigs in the immunobiotic TUA4408L-fermented okara feed group had significantly higher expression levels of TGF-β than the control groups (Figure 6C).

## 4. Discussion

Okara, a byproduct from the soymilk and tofu industry, was recently proposed as an economical and environmentally friendly feed option for pigs [18,25]. However, the unpalatable and insoluble nature of okara requires the transformation of this product before being used as a suitable feed. In this regard, it was proposed that fermentation processes might improve the nutritional value of okara by releasing nutrients and changing its physicochemical properties [18]. Moreover, anti-nutritional factors of okara might be eliminated by fermentation [26]. Hence, the primary aim of the present study was to propose a new immunobiotic feed by using the potent immunoregulatory strain *L. delbrueckii* subsp. *delbrueckii* TUA4408L and the soymilk by-product, okara, and to determine the potential of this functional fermented feed to serve as a suitable stimulator of the growth, immune-health, and productivity of pigs. Here, we provide original in vivo information concerning the beneficial effects of the immunobiotic TUA4408L-fermented okara feed by demonstrating that its administration significantly increased growth performance, meat quality, and immune health of piglets.

The slow growth of pigs within a batch is usually a cause of the non-efficient use of the growing and fattening facilities, significantly reducing the pig producer’s income. Our group previously demonstrated the ability of *Lactobacillus jensenii* TL2937 to improve piglet growth performance and productivity [19]. In fact, piglets fed the TL2937 strain reached the body weight for slaughter earlier than controls with a difference of 4 weeks. Similarly, the administration of the immunobiotic TUA4408L-fermented okara feed allowed pigs to reach slaughter weight in week 20, 4 weeks earlier than the control group. This could undoubtedly represent an advantage for producers, due to the economic benefit of reducing barn occupancy and feed administration 4 weeks earlier.

The administration of the immunobiotic TUA4408L-fermented okara feed to pigs also increased the intramuscular fat content of their meat. The intramuscular fat of porcine meat is closely correlated with meat quality, especially in terms of eating quality because it is related to tenderness, flavor, and juiciness [27]. In line with this finding, when the hardness of the meat was measured by the one-byte method, it was significantly lower in the immunobiotic TUA4408L-fermented okara feed group than in the controls. The intramuscular fat is also critical to the meat’s nutritional value [28,29]. It was shown that the fatty acid profiles of fat muscle play a key role in meat nutritional quality. The presence of polyunsaturated fatty acids is considered beneficial to the prevention of cardiovascular disease in humans [30,31] as well as the development of insulin resistance and metabolic disorders [32]. The fatty acid composition of porcine meat can be regulated by nutritional interventions [33,34]. Accordingly, we observed here that the administration of the immunobiotic TUA4408L-fermented okara feed to pigs induced changes in the fatty acid profile of meat by decreasing saturated and increasing unsaturated fatty acids. This effect could be related to the changes in the meat quality because softer meats have been associated with an increase in total unsaturated fatty acids [27]. In addition, the changes in lipid composition induced by the immunobiotic TUA4408L-fermented okara feed contributed to a meat that would be healthier for human consumption.

It should be noted that the changes in the composition of fatty acids of the pork intramuscular fat induced by the immunobiotic TUA4408L-fermented okara feed were modest when compared with those induced by other dietary interventions [35,36]. Drastic changes in the composition of fatty acids of pork intramuscular fat are induced only with diets that aim for remarkable quantitative and/or qualitative changes in the source of lipids. Moreover, these changes have been associated with both beneficial (nutritional) and adverse effects. In fact, in addition to its nutritional value, the fatty acid composition also influences the oxidative stability of meat [37]. Recently, it was shown that an olive by-product combined with betaine, magnesium, and vitamin E is capable of modifying pigs’ performance and meat quality characteristics [35]. The researchers supplemented the basal conventional diet of pigs with the mix mentioned before (high-dose diet) or half-dose of each compound (low-dose diet). The pigs’ performance and carcass yield or intramuscular fat were not changed by dietary supplementation with either of the two additive-mixtures. However, the high-dose diet was able to significantly increase the proportion of polyunsaturated fatty acids whereas the proportion of total monounsaturated fatty acids was lower. No changes between the control and half-dose diet were detected when the composition of fatty acids in intramuscular fat was compared [35]. Of note, the specific fatty acid composition of meat from pigs in the high-dose diet group resulted in meat that was more susceptible to oxidation. Hence, dietary intervention with the immunobiotic TUA4408L-fermented okara feed could induce a modest nutritional improvement of the meat in terms of its fatty acid composition, without modifying its susceptibility to oxidation.

To the best of our knowledge, only one study has evaluated the potential beneficial effects of probiotic-fermented okara in pigs. Fermented okara prepared with a complex mixture of commercial probiotics that included *L. plantarum*, *B. subtilis*, and *S. cerevisiae* in 1:1:1 proportions was able to enhance the average daily weight gain and final body weight of pigs when compared to control animals [18]. Furthermore, fermented okara administration increased the intramuscular fat content of *longissimus thoracis* muscle, but parameters used in evaluating the meat quality were not statistically different when treated animals were compared to control pigs. These and our own results indicate that the specific strains of microorganisms used to ferment okara may be important to improve the quality of the meat. Thus, the proper selection of probiotic microorganisms to be used to ferment okara could allow not only increasing the pig’s productivity but also the quality of the meat.

In addition to rationally selecting probiotic strains to develop okara-based feeds, it is of great importance to investigate the mechanisms involved in their beneficial effects in pigs. It is necessary to determine if the differential effect on growth performance and meat quality induced by probiotic okara-based feeds is directly correlated with the transformation of the feed substrate and the subsequent bioavailability of nutrients, or if it is an indirect effect associated with the improvement of the intestinal microbiota composition and/or the immune status of the porcine host. Regarding this last point, the studies carried out in this work demonstrated the ability of the immunobiotic TUA4408L-fermented okara feed to beneficially modulate both the intestinal microbiota and immunity of pigs, providing for the first time clues about the mechanisms that could be involved in the improvement of pig health with a probiotic-fermented okara.

It was reported that the gut microbiota, through their metabolic activities and immunoregulatory functions, significantly influence the health of pigs and therefore, qualitative and/or quantitative changes in the intestinal microbial populations can significantly affect whole-body growth throughout the productive life cycle of these animals [3,38]. The most important modifications in the composition of the intestinal microbiota of pigs are produced during early life when piglets need to adapt to new diets and environments. Hence, the intestinal microbiota during the earlier stages of pig growth require special consideration when viewed in the context of pig production [3]. Studies of porcine intestinal microbiota have demonstrated the importance of *Lactococcus* and *Lactobacillus* in the early life of piglets [39]. In an interesting recent work that aimed to evaluate intestinal microbiota markers that were associated with low versus normal birth weight in pigs and their correlation with the subsequent good or poor average daily gain, it was found that piglets with optimal growth performance had a significantly higher abundance of *Lactobacillus* earlier after birth [40]. Of note, the improvement of beneficial intestinal bacteria can be achieved with dietary interventions. In this regard, it was reported that dietary fibers such as alfalfa are able to beneficially influence the composition of pigs’ gut microbiota and their growth performance [41]. The alfalfa meal treatment significantly enhanced the relative abundance of *Firmicutes* and decreased *Tenericutes* in the jejunum of pigs. Furthermore, the work found that the dietary intervention increased the relative abundance of *Lactococcus*, *Enterococcus*, *Paenibacillus*, *Bacillus*, and *Oceanobacillus*, which have been associated with the inhibition of pathogen growth and modulation of the immune system [41].

Fecal microbiota transplantation and the administration of probiotic microorganisms also have been proposed as efficient treatments to beneficially modulate the porcine intestinal microbiota. Early intervention with fecal microbiota from gestating sows combined with probiotic strains of the species *Clostridium butyricum* and *Saccharomyces boulardii* was shown to be capable of decreasing opportunistic pathogens, while enhancing the relative abundance of beneficial bacteria in the gut of pigs [20,42]. In fact, the work reported a significant increase in *Lactobacillales* and *Coriobacteriales* in treated pigs when compared to controls, which was associated with improved development of the immune system. On the other hand, the administration of the probiotic strain *L. plantarum* ZLP001 was also shown to increase *Lactobacillales* in the porcine gut and reduce *Clostridiales*, which are often associated with epithelial inflammation [43]. Of note, the modulation of gut microbiota induced by *L. plantarum* ZLP001 was associated with a strengthening of epithelial defense functions because increased expression of claudin-1, occludin, and ZO-1 as well as the host defense peptides pBD2, PG1-5, and pBD2 was detected in the intestinal epithelium of treated pigs. Moreover, the changes induced by the ZLP001 strain significantly counteracted the increase in gut permeability induced by ETEC challenge. In line with these previous findings, we demonstrated here that the immunobiotic TUA4408L-fermented okara feed was capable of enhancing the abundance of both *Lactococcus* and *Lactobacillus* in the intestine of pigs. The immunobiotic TUA4408L-fermented okara feed was thus capable of improving intestinal microbiota composition and through this effect, it could help reinforce the intestinal barrier.

In addition to their direct beneficial effects on nutrient availability and competition with pathogens, intestinal microorganisms and probiotics can exert positive effects on the host through their interaction with the immune system. It was reported that a diet supplemented with a probiotic mixture including strains of the species *Clostridium butyricum*, *Bacillus subtilis*, and *Bacillus licheniformis* improved the growth performance of pigs and that this beneficial effect was associated with the protection of the intestinal epithelium against harmful inflammation [44]. In fact, the probiotic treatment significantly reduced the serum and intestinal levels of the inflammatory factors TNF-α, IL-1β, and IL-6. Similarly, the administration of *L. plantarum* JDFM LP11 to pigs differentially regulated the expression of inflammatory factors in the intestinal mucosa [45]. RNA sequencing analysis of the ileum of probiotic-treated animals found a significant reduction in the expression of inflammatory factors *BPI, RSAD2, SLPI, LUM, OLFM4, DMBT1*, and *C6*. Probiotic treatments have also been shown to be effective in protecting against infection and the harmful inflammatory response produced by Gram negative pathogens such as ETEC [19,43,46]. Recently, efforts have been made to understand the molecular mechanisms involved in the protection induced by probiotic microorganisms against intestinal inflammation in pigs, and it has been found that negative regulators of TLR signaling pathways could play a key role. Transcriptomic studies of the small intestinal epithelium of conventional versus germ-free neonatal piglets found higher levels of NF-κBIA and Tollip, two proteins associated with the negative regulation of TLR signaling pathways, in colonized versus germ-free animals [47]. Moreover, the presence of the intestinal microbiota was associated with a down-regulation of GATA1 in the gut epithelium. We also demonstrated previously that the immunobiotic strain *L. jensenii* TL2937 improved the growth performance and productivity of pigs [19] and that this effect was associated with a differential modulation of intestinal inflammation. The TL2937 strain had a high capacity to reduce pro-inflammatory factor expression in PIE cells in response to ETEC or LPS challenges [48,49]. This effect correlated with the ability of *L. jensenii* TL2937 to inhibit NF-kB and MAPK signaling pathways through up-regulation of the negative regulators MKP-1, A20, and Bcl-3. In addition, we demonstrated that the TL2937 strain enhanced the expression of negative regulators of TLRs in porcine antigen-presenting cells [50,51]. Of the six regulators tested, SIGIRR, A20, and IRAK-M mRNA were up-regulated in porcine CD172a^+^ antigen-presenting cells stimulated with *L. jensenii* TL2937 and subsequently challenged with LPS or ETEC. In line with those findings, we demonstrated here that the immunobiotic TUA4408L-fermented okara feed significantly reduced the levels of blood CRP and the granulocyte/lymphocyte ratio in pigs, indicating a beneficial regulation of inflammation. Furthermore, when immune factor expressions were evaluated in the intestinal mucosa, it was found that the immunobiotic TUA4408L-fermented okara feed treatment reduced the expression of IL-6 and IL-8 while it increased the expression of TGF-β. These observations are in agreement with our in vitro studies demonstrating the capacity of *L. delbrueckii* subsp. *delbrueckii* TUA4408L to differentially modulate the innate immune response of PIE cells to the challenge with ETEC by reducing the activation of NF-κB, ERK-MAPK, and p38-MAPK pathways through up-regulation of the expression of SIGIRR and Tollip [15].

Several studies have reported that immunobiotics are capable of modulating the intestinal immune system to enhance protection against infections and regulate inflammation. Of note, those effects can be achieved simultaneously with the same immunobiotic strains because of their ability to differentially regulate inflammatory and anti-inflammatory cytokines [50,52]. We have shown consistently that the oral administration of some immunobiotic strains including *Lacticaseibacillus casei* CRL431, *Lactiplantibacillus plantarum* CRL1506, and *Lacticaseibacillus rhamnosus* CRL1505 improved the production of IFN-γ, IFN-β, and IL-10 in the gut and reduced the levels of IL-8 and MCP-1 in the context of intestinal bacterial and viral infections [22,53,54]. The differential cytokine profile induced by immunobiotics was translated to enhanced pathogen clearance and reduced inflammatory-mediated damage. The results obtained in this work allow us to speculate that the immunobiotic TUA4408L-fermented okara feed would induce this immunoregulatory benefit in pigs. In our hands, pigs treated with the immunobiotic TUA4408L-fermented okara feed showed a significant reduction in the presence of pathogenic *E. coli* and lower levels of the markers of inflammation. It is tempting to speculate that the ability of *L. delbrueckii* subsp. *delbrueckii* TUA4408L to functionally modulate the porcine intestinal epithelium [15,16] could have a key role in the immunological changes observed in pigs. The immunobiotic TUA4408L-fermented okara feed increased the expression of TGF-β in pig intestine. It was established that the immune factors derived from IECs are capable of conditioning the intestinal immune cells, especially DCs, to differentially secrete cytokines in response to commensal microbes or pathogens, thereby promoting the development of different T cells effectors [55]. In fact, the conditioning of monocyte-derived DCs with IEC supernatants confers on DCs the capacity to produce large amounts of IL-10, which is attributable, at least in part, to the release of IEC-derived factors such as TGF-β [56]. In line with these findings, we demonstrated previously that the immunobiotic strain *L. jensenii* TL2937 was able to significantly up-regulate TGF-β expression in porcine IECs and, through this mechanism, to improve the synthesis of IL-10 in CD172a^+^CD11R1^high^ and CD172a^+^CD11R1^−^ porcine intestinal antigen-presenting cells [19,50]. Simultaneously, the direct interaction of the TL2937 strain with CD172a^−^CD11R1^low^ cells increased their production of IFN-γ [19,50]. Because the feeding of pigs with *L. jensenii* TL2937 reduced the recovery of pathogenic *E. coli* and the levels of inflammation markers, it could be speculated that the immunobiotic TUA4408L-fermented okara feed would be also capable of modulating porcine intestinal antigen-presenting cells. Evaluating the effect of *L. delbrueckii* subsp. *delbrueckii* TUA4408L and the immunobiotic TUA4408L-fermented okara feed on porcine intestinal antigen-presenting cells could be of great value to understanding the mechanisms of their immunological benefits, and for this reason, we propose that such studies be carried out in the near future.

In conclusion, our in vivo studies demonstrated that the immunobiotic TUA4408L-fermented okara feed significantly increased piglet growth performance and meat quality. These positive effects were associated with the ability of the immunobiotic TUA4408L-fermented okara feed to beneficially modulate both the intestinal microbiota and immunity of pigs. The immunobiotic feed improved the abundance of the beneficial bacteria *Lactobacillus* and *Lactococcus* in the gut of pigs, reduced blood markers of inflammation, and differentially regulated the expression of inflammatory and regulatory cytokines in the intestinal mucosa. These findings indicate that the immunobiotic TUA4408L-fermented okara feed could be an economical and environmentally friendly option to improve the growth performance and immune health of pigs.

## Figures and Tables

**Table 1:** Fatty acid composition of the subcutaneous fat of the loin core. The control group was fed only the different balanced conventional diets without antimicrobials, while the SM control group and the TUA4408L group were fed balanced conventional diets with supplemental soymilk or the immunobiotic TUA4408L-fermented okara, respectively.

Fatty Acid	Control	SM Control	TUA4408L
Myristic acid	2.1	1.1 *	1.0 *
Palmitic acid	27.6	24.9	23.9
Palmitoleic acid	1.2	1.3	1.1
Stearic acid	15.6	14.9	10.9 *
Oleic acid	40.2	41.0	45.3
Linoleic acid	8.2	10.9	15.9 *
Linolenic acid	0.8	1.0	0.8
Saturated fatty acids	45.3	40.9	35.8 *
Unsaturated fatty acid	50.4	54.2	63.1 *
Monounsaturated fatty acids	41.4	42.3	46.4
Δ9index	0.7	0.7	0.8

Asterisks (*) indicate statistical differences between the treated and control groups (*p* < 0.05).

**Table 2:** Clinical symptoms in pigs registered during the studied period. The control group was fed only the different balanced conventional diets without antimicrobials while the SM control group and the TUA4408L group were fed balanced conventional diets with supplemental soymilk or the immunobiotic TUA4408L-fermented okara, respectively.

Group	Registered Diseases
Control	Drowning, cough, mycoplasma pneumonia, eczema, diarrhea, kidney disease, liver disease
SM Control	Drowning, cough, mycoplasma pneumonia, diarrhea
TUA4408L	Mild diarrhea

## Data Availability

The data presented in this study are available on request from the corresponding author.

## Supplementary Materials

The following are available online at https://www.mdpi.com/article/10.3390/microorganisms9050921/s1, Table S1: Nutritional composition of diets administered to piglets.
