# Supplementary material for: Immunobiotic Feed Developed with Lactobacillus delbrueckii subsp. delbrueckii TUA4408L and the Soymilk By-Product Okara Improves Health and Growth Performance in Pigs
Source: Microorganisms. 2021 Apr 25;9(5):921. doi: 10.3390/microorganisms9050921 (PMC8145491; doi:10.3390/microorganisms9050921)
Supplement: Supplementary file 1 [file microorganisms-09-00921-s001.zip › microorganisms-1168937-SI.pdf]

Supplementary Table S1. Nutritional composition of diets administered to piglets.

Nutrient (%)	**After weaning to 30 kg**	**From 30 kg to 70 kg**	**From 70 kg to 120 kg**
**Protein**	More than 18.0	More than 15.0	More than 14.0
**Fat**	Moe than 3.5	More than 4.0	More than 2.5
**Fiber**	Less than 4.0	Less than 6.0	Less than 5.0
**Mineral**	Less than 8.0	Less than 8.0	Less than 8.0
**Calcium**	More than 0.75	More than 0.5	More than 0.5
**Phosphorus**	More than 0.60	More than 0.4	More than 0.4
**Total digestible nutrients**	More than 80.0	More than 78.0	More than 77.0
